# Metacognitive model of suicidality: a study of Iranian inpatients

**DOI:** 10.3389/fpsyt.2026.1766691

**Published:** 2026-05-29

**Authors:** Zahra Asgari, Kaveh Qaderi Bagajan, Matin Khanpayeh, Mahnaz Abdi, Lena Marie Hensel, Tobias Teismann

**Affiliations:** 1 Department of Counseling, Faculty of Education and Psychology, University of Isfahan, Isfahan, Iran; 2 Department of Clinical and Health Psychology, Shahid Beheshti University Tehran, Tehran, Iran; 3 Department of Clinical Psychology, Kurdistan University of Medical Science, Sanandaj, Iran; 4 Neurosciences Research Center, Research Institute for Health Development, Kurdistan University of Medical Sciences, Sanandaj, Iran; 5 Mental Health Research and Treatment Center, Ruhr-Universität Bochum, Bochum, Germany; 6 DZPG (German Center for Mental Health), partner site Bochum/Marburg, Bochum, Germany

**Keywords:** metacognitions, metacognitive model, rumination, suicidal ideation, suicide attempts

## Abstract

**Background:**

The metacognitive model of suicidality proposes that positive metacognitions about suicide activate suicide-specific rumination, which in turn leads to the activation of negative metacognitions about suicide and an escalating aggravation of suicidal ideation/behavior. Initial studies support the model assumptions. However, investigations in highly burdened inpatient samples as well as studies in non-Western samples are missing by now.

**Methods:**

A total of 209 Iranian psychiatric inpatients (56.9% female; age *M* = 31.14, *SD* = 11.04) took part in a cross-sectional assessment. Self-report measures to assess suicidal ideation/behavior, depressive symptoms, suicide-specific rumination, and metacognitions about suicide were used.

**Results:**

Positive metacognitions about suicide were associated with suicide-specific rumination. Suicide-specific rumination was associated with negative metacognitions about suicide. Suicidal thoughts were positively and positive metacognitions about suicide were negatively associated with lifetime suicide attempts.

**Conclusion:**

The results support assumptions of the metacognitive model of suicidality and underscore the importance of metacognitions about suicide and suicide-specific rumination in understanding the suicidal process.

## Background

Psychiatric inpatients are at a substantial risk of suicide, particularly during the first week following admission and the first four weeks after discharge (1, 2). Key risk factors for inpatient suicide include a history of lifetime self-harm, suicidal ideation and depression (3–5), and this pattern appears comparable across both Western and Asian populations (6, 7). Beyond completed suicides, inpatients all over the world also experience considerable burden from suicidal ideation and lifetime suicide attempts (8–10). Finally, with regard to the treatment of suicidal patients, a theoretical understanding of suicidal crises - specifying which factors should be prioritized in risk assessment, crisis intervention, and psychotherapy - is of particular importance (cf. 11). Building on findings suggesting that (suicide-specific) rumination may constitute a highly relevant risk factor for both suicidal ideation and suicide attempts, several promising contributions toward a metacognitive conceptualization have recently been published (12–14).

Wells’ metacognitive model (15) has become a key framework for understanding and treating emotional disorders (16–19). In this model, Wells (15) highlights the central role of the so-called Cognitive Attentional Syndrome (CAS) as a core characteristic of psychological disorders. The CAS consists of three elements (1.) perseverative thinking in the form of rumination and worry, (2.) attentional monitoring of threat-related stimuli, and (3.) maladaptive coping strategies such as thought suppression. It is believed that the CAS disrupts self-regulation by hindering emotional processing, locking cognition onto perceived threats, reinforcing negative appraisals and beliefs, and obstructing corrective learning experiences. Wells (15) states that the CAS is governed by mistaken beliefs about thinking. These include positive metacognitive beliefs about the usefulness or necessity of rumination, threat monitoring, and thought control strategies (e.g., ‘Rumination will help me gain insight’), as well as negative metacognitive beliefs concerning the uncontrollability and danger of thoughts and reasoning processes (e.g., ‘Rumination is uncontrollable’). In addition to the general metacognitive model, Wells (15) clarified the specific interplay between metacognitions and the Cognitive Attentional Syndrome (CAS), in various disorder-specific models.

Building on this work, Forkmann et al. (12) recently presented the metacognitive model of suicidality. This model proposes that thoughts related to death and dying trigger positive metacognitions (e.g., ‘Suicidal thoughts show me a way out’), which in turn promote sustained suicide-related rumination. Once the CAS is activated, individuals may experience an increase in rumination, an attentional fixation on suicidal thoughts and unsuccessful attempts to suppress these thoughts. This in turn may lead to the activation of negative metacognitions concerning the uncontrollability and harmfulness of suicidal thoughts (cf. 20). Ultimately, the activation of negative metacognitions may not only intensify the CAS, but also contribute to the worsening of suicidality, leading to suicidal planning, the development of suicidal intent, and suicidal behavior. In line with the theoretical assumptions, Forkmann et al. (12) found that positive suicide-related metacognitions predicted suicide-specific rumination (beyond the effect of suicidal ideation) and that suicide-specific rumination predicted negative suicide related metacognitions (beyond the effect of suicidal ideation) in German participants with lifetime suicidal ideation. In another sample of German participants with lifetime suicidal ideation, Forkmann et al. (13) found positive suicide-related metacognitions to predict suicide-specific rumination as well as attentional fixation on suicide; suicide-specific rumination, attentional fixation on suicide and thought control strategies were shown to predict negative suicide related metacognitions. In a final study, Forkmann et al. (14) investigated German patients receiving inpatient psychiatric treatment. In this study both positive and negative suicide-related metacognitions predicted suicide-specific rumination. Furthermore, suicide-specific rumination, attentional fixation as well as positive and negative suicide-related metacognitions differentiated between lifetime suicide attempters and non-attempters (12, 13). Further studies from Europe and Asia have also demonstrated that general - that is, non–suicide-related -, negative metacognitions (about the danger and uncountability of repetitive negative thoughts) are associated with suicidal ideation (21–23), and that negative metacognitive beliefs (24, 25) as well as positive metacognitive beliefs (24) are stronger in those with lifetime suicide attempts compared to non-attempters. Although various facets of the CAS have been studied, rumination has been the most frequently identified as significant (26). Specifically, suicide-specific rumination - rumination about death and/or suicide - has been linked to suicidal ideation in American (27, 28), German (29, 30) and Iranian participants (31). Furthermore, suicide-specific rumination was found to prospectively predict suicide intent and suicide planning (32, 33) as well as to differentiate between (multiple/single) suicide attempters and non-attempters (27, 28, 30, 34). Taken together, suicide-specific rumination seems to be of special importance in understanding suicidal escalations.

However, the association between metacognitions about suicide, suicide-specific rumination and suicidal thoughts has only once been investigated in a sample of highly burdened psychiatric inpatients and no study by now has been conducted in a non-Western society - and this despite substantial cultural differences in suicidal ideation and behavior (35) as well as attitudes against suicide (36, 37) and the fact that it cannot be assumed that theoretical models for understanding suicidal crises are equally applicable across different cultures (38).

Given this background, the purpose of the present study was threefold: (1) First, we aimed to investigate whether positive suicide-related metacognitions are associated with suicide-specific rumination (after controlling for lifetime suicide attempts, concurrent depression and suicidal ideation) in a sample of Iranian inpatients. (2) Second, we aimed to investigate whether suicide-specific rumination is associated with negative suicide-related metacognitions (after controlling for lifetime suicide attempts, concurrent depression and suicidal ideation). (3) Third, we examined whether suicide-specific rumination as well as positive and negative suicide-related metacognitions are associated with lifetime suicide attempts (after controlling for concurrent depression and suicidal ideation). In line with the metacognitive model of suicidality and previous studies on Western samples (12–14) we hypothesized, that positive suicide-related metacognitions predict suicide-specific rumination and suicide-specific rumination predicts negative suicide-related metacognitions in an adult sample of Iranian inpatients. Finally, we hypothesized that metacognitions about suicide as well as suicide-specific rumination are associated with lifetime suicide attempts (12).

## Materials and methods

### Participants and procedure

In total, *N* = 209 participants (*n* = 119 female; 56.9%; age *M* = 31.14, *SD* = 11.04) took part in a single assessment between February and April 2025. In total, 191 participants (91.4%) indicated suicidal ideation within the last four weeks (SIBS ≥ 1) and 65 participants (31.1%) indicated that they had attempted suicide at least once in their lifetime. The most common primary diagnoses according to the DSM 5^®^ were Borderline Personality Disorder (23.4%, *n* = 49), Adjustment Disorder (18.7%, *n* = 39), Bipolar Disorder (18.2%, *n* = 38), Major Depressive Disorder (13.4%, *n* = 28), Somatic Symptom Disorder (10.0%, *n* = 21) and Obsessive Compulsive Disorder (9.1%, *n* = 19). Data were collected at the Qods Psychiatric Hospital in Sanandaj, Iran. In order to take part in the study, participants had to be at least 18 years old and give their consent to participation at the beginning of the study. Prior to assessments, participants were informed about the purpose of the study, the voluntary nature of their participation, data storage and security. They gave written informed consent before participating. All procedures were in accordance with the Helsinki Declaration as revised 1989. The study was approved by the Ethics Committee of the research center in Tehran, Iran (IR.ATU.REC.1399.093).

### Measures

#### Scales for the assessment of suicide-related metacognitions

The SSM assess positive suicide-related metacognitions (e.g., “Suicidal thoughts show me a way out”) with 5 items and negative metacognitions (e.g., “Suicidal thoughts are uncontrollable”) with 9 items. All items are answered on a 4-point scale ranging from 0 (“I do not agree”) to 3 (“I very much agree”). Internal consistency in terms of Cronbach’s α of the two scales of the SSM were shown to be good: α*_NM_* = 0.90 and α*_PM_* = 0.83 (12). The SSM was translated to Farsi by using a translation-backtranslation procedure (39). Internal consistency of the translated version was good in the current sample: α*_NM_* = 0.95 and α*_PM_* = 0.88.

#### Perseverative thinking about suicide questionnaire

The PTSQ (12) is modeled after the Perseverative Thinking Questionnaire (PTQ; 40) and assesses suicide specific rumination with four items (e.g., “I get stuck on thoughts about suicide and can`t move on”). All items are to be answered on a 5-point scale ranging from 0 (“never”) to 4 (“almost always”). The scale has been shown to have high internal consistency (Cronbach’s α = 0.93) and a unidimensional factor structure (29). The PTSQ was translated to Farsi by using a translation-backtranslation procedure (39). In the current sample, the translated scale showed high internal consistency: *α* = 0.93.

#### Suicide ideation and behavior scale

The SIBS (41) comprises six items assessing suicidal ideation, suicidal intent, suicidal impulses and suicide planning within the last 4 weeks. Items are rated on a 6-point Likert-type scale (0= “never”, 5=“many times a day”). Three further items assess the occurrence of a suicide attempt within the last four weeks as well as occurrence/frequency of lifetime suicide attempts. Excellent internal consistency has been demonstrated in an Iranian sample: α=.88 (42). Internal consistency was α=.92 in the current sample.

#### Patient health questionnaire

The Patient Health Questionnaire (PHQ-9) (43) assesses symptoms of depression (e.g., lack of interest, feeling down, poor appetite) with nine items (e.g., “Over the last two weeks, how often have you been bothered by any of the following problems: Little interest or pleasure in doing things?”) that are rated on a 4-point Likert-type scale (0=“not at all”, 3=“nearly every day”). Internal consistency was shown to be good in the Iranian validation study: α ≥.91 (44). In line, internal consistency was good in this sample: α=.91. The suicide item (Item 9) was excluded from the PHQ-sum score in all analyses to avoid overlapping and thus part-whole correlations with other suicide-focused questionnaires. The PHQ-8 (leaving out Item 9) has been shown to have excellent validity and reliability and has been recommended for screening purposes (45).

### Data analysis

All analyses were conducted using SPSS 29. Descriptive statistics of all measures as well as Pearson correlations were calculated. In addition, exploratory analyses were conducted to examine the extent to which the different diagnostic groups differ with respect to the central study variables. To this end, one‐way ANOVAs with Tukey HSD comparisons were performed. To test the metacognitive model of suicidality, a series of regression analyses were performed. First, it was analyzed whether positive suicide-related metacognitions are associated with suicide-specific rumination. Second, it was analyzed whether negative suicide-related metacognitions are associated with suicide-specific rumination. In both analyses we controlled for concurrent depression, suicidal ideation and lifetime suicide attempts. There was no violation of the multicollinearity assumption as all values of tolerance were >.25, and all variance inflation factor values were <5. Assuming a medium-sized effect (*f*^2^ = 0.15), an alpha error level of 5%, four predictors and a sample size of *N* = 209, the test power was 1-ß=0.99. Finally, a series of separate logistics regression analyses as well as a multiple logistic regression analysis was used to examine the variables’ relative contribution to the prediction of the presence of a lifetime suicide attempt.

## Results

Descriptive statistics and intercorrelations of all measures used in this study are depicted in **Table 1**. **Table 2** presents the results of exploratory ANOVAs comparing the different diagnostic categories. The findings indicate that patients with a primary diagnosis of Borderline Personality Disorder or Major Depression exhibit higher levels of depression, suicidal ideation, negative suicide-related metacognitions, and suicide-specific rumination than patients with other primary diagnoses.

**Table 1:** Descriptive statistics and intercorrelations (*N* = 209).

	*M* (*SD*)	1	2	3	4	5
1. SIBS	7.46 (5.84)		.			
2. PTSQ	5.12 (3.86)	.83**				
3. SSM-NM	12.36 (6.97)	.76**	.79**	.		
4. SSM-PM	8.43 (3.72)	.44**	.52**	.51**		
5. PHQ-8	12.16 (5.54)	.66**	.68**	.69**	.38**	–

**p ≤.001; PHQ, Patient Health Questionnaire; PTSQ, Perseverative Thinking about Suicide Questionnaire; SIBS, Suicidal Ideation and Behavior Scale; SSM-PM, Positive Metacognitions Scale of the Scales for the Assessment of Suicide-related Metacognitions; SSM-NM, Negative Metacognitions Scale of the Scales for the Assessment of Suicide-related Metacognitions.

**Table 2:** Group differences in relation to primary diagnosis.

Variables	BPS *M* (*SD*)	MDE *M* (*SD*)	BD *M* (*SD*)	AD *M* (*SD*)	SSD *M* (*SD*)	OCD *M* (*SD*)	F-Statistics	Group comparison
PHQ-8	16.20 (2.98)	19.07 (3.01)	7.05 (3.11)	11.5 (3.66)	5.00 (2.68)	9.84 (3.37)	*F*(5, 188) = 85.78**	MDE > BPS > AD, OCD > BD, SSD
SIBS	11.08 (5.17)	13.71 (6.68)	3.68 (3.55)	5.69 (3.07)	2.57 (3.07)	4.42 (3.68)	*F*(5, 188) = 30.82**	MDE, BPS > AD, BD, SSD, OCD
PTSQ	7.08 (3.53)	9.10 (4.13)	2.63 (2.23)	4.38 (2.82)	1.66 (1.68)	3.63 (2.16)	*F*(5, 188) = 26.28**	MDE, BPS > AD ≥ BD, OCD, SSD
SSM-PM	10.02 (2.99)	9.00 (3.95)	7.02 (3.12)	9.15 (3.03)	5.23 (4.83)	7.94 (3.47)	*F*(5, 188) = 7.46**	BPS ≥ MDE, OCD, AD, BD ≥ SSD
SSM-NM	16.48 (5.81)	18.17 (5.81)	7.50 (5.12)	11.66 (5.65)	6.23 (5.81)	9.21 (4.58)	*F*(5, 188) = 22.74**	MDE, BPS > AD, OCD ≥ BD, SSD

**p ≤.001; AD, Adjustment Disorder; BD, Bipolar Disorder; BPS, Borderline Personality Disorder; MDE, Major Depression Episode; OCD, Obsessive Compulsive Disorder; PHQ, Patient Health Questionnaire; PTSQ, Perseverative Thinking about Suicide Questionnaire; SIBS, Suicidal Ideation and Behavior Scale; SSD, Somatic Symptom Disorder; SSM-PM, Positive Metacognitions Scale of the Scales for the Assessment of Suicide-related Metacognitions; SSM-NM, Negative Metacognitions Scale of the Scales for the Assessment of Suicide-related Metacognitions.

### Associations between suicide-related metacognitions and suicide-specific rumination

It was first tested whether positive suicide-related metacognitions are associated with suicide-specific rumination. In fact, positive suicide-related metacognitions were significantly associated with suicide-specific rumination, after controlling for current depression, suicidal ideation and lifetime suicide attempts (see **Table 3**).

**Table 3:** Prediction of suicide-specific rumination (*N* = 209).

		Model 1				Model 2		
	B	T	Beta	p	B	T	Beta	p
PHQ-8	.162	4.70	.23	.001	.143	4.26	.20	.001
SIBS Lifetime Suicide Attempts	.454 -.19	12.98 -.55	.69 -.02	.001 .583	.408 .044	11.47 .12	.62 .01	.001 .901
SSM-PM	-	-		-	.172	4.10	.16	.001
Model		Adj. R^2^ = .718 *F*(3, 205)=177.88 *p* <.001				Adj. R^2^ = .739 *F*(4, 205)=147.93 *p* <.001		
Change in R^2^		0.722 *p* <.001				0.021 *p* <.001		

PHQ, Patient Health Questionnaire; SIBS, Suicidal Ideation and Behavior Scale; SSM-PM, Positive Metacognitions Scale of the Scales for the Assessment of Suicide-related Metacognitions.

In a second regression analysis, it was tested whether suicide-specific rumination was associated with negative suicide-related metacognitions. Results showed that suicide-specific rumination was significantly associated with negative suicide-related metacognitions, after controlling for current depression, suicidal ideation and lifetime suicide attempts (see **Table 4**).

**Table 4:** Prediction of negative suicide-related metacognitions (*N* = 209).

		Model 1				Model 2		
	B	T	Beta	p	B	T	Beta	p
PHQ-8	.415	5.93	.33	.001	.300	3.95	.24	.001
SIBS Lifetime Suicide Attempts	.683 -.912	9.59 -1.24	.57 -.06	.001 .216	.358 -.770	4.33 -1.12	.24 -.05	.001 .216
PTSQ	-	-		-	.716	5.36	.39	.001
Model		Adj. R^2^ = .640 *F*(3, 205)=124.50 *p* <.001				Adj. R^2^ = .683 *F*(4, 204)=113.17 *p* <.001		
Change in R^2^		0.646 *p* <.001				0.044 *p* <.001		

PHQ, Patient Health Questionnaire; PTSQ, Perseverative Thinking about Suicide Questionnaire; SIBS, Suicidal Ideation and Behavior Scale.

### Association between suicide-related metacognitions, suicide-specific rumination and lifetime suicide attempts

Depression, suicidal ideation, negative suicide-related metacognitions as well as suicide specific rumination were associated with a lifetime suicide attempt in separate logistic regression analyses (see **Table 5**). In a multiple logistic regression model (Nagelkerkes R^2^ = .384) with depression, suicidal ideation, positive metacognitions, negative metacognitions and suicide-specific rumination, only suicidal ideation and positive metacognitions were associated with the presence of a lifetime suicide attempt (see **Table 5**).

**Table 5:** Results from separate and multiple logistic regression analyses predicting a lifetime suicide attempt.

Baseline variable	Lifetime suicide attempt (*n* = 65) vs. no attempt (*n* = 144)			
	Separate^a^		Multiple^b^	
	OR (95% CI)	*p*	OR (95% CI)	*p*
PHQ-8	1.19 (1.11-1.27)	.001	1.08 (0.97-1.06)	.178
SIBS	1.25 (1.16-1.33)	.001	1.26 (1.12-1.41)	.001
SSM-PM	1.07 (0.98-1.16)	.113	0.87 (0.76-0.99)	.039
SSM-NM	1.13 (1.08-1.19)	.001	0.97 (0.89-1.06)	.589
PTSQ	1.28 (1.17-1.40)	.001	1.03 (0.86-.1.24)	.698

PHQ, Patient Health Questionnaire; PTSQ, Perseverative Thinking about Suicide Questionnaire; SIBS, Suicidal Ideation and Behavior Scale; SSM-NM, Negative Metacognitions Scale of the Scales for the Assessment of Suicide-related Metacognitions; SSM-PM, Positive Metacognitions Scale of the Scales for the Assessment of Suicide-related Metacognition.

^a^ Separate logistic regressions, each with a single predictor variable.

^b^ Multiple logistic regression model with all predictor variables.

## Discussion

In the present study, the metacognitive model of suicidality was examined for the first time first time in a non-Western sample of highly stressed psychiatric inpatients. There were three main findings: (1.) Positive suicide-related metacognitions was associated with suicide-specific rumination. (2.) Suicide-specific rumination was associated with negative metacognitions about suicide. (3.) Suicidal ideation and positive suicide-related metacognitions were associated with lifetime suicide attempts.

In line with the theoretical assumptions of the metacognitive model of suicidality (12) positive suicide-related metacognitions were associated with suicide-specific rumination and suicide-specific rumination was associated with negative suicide-related metacognitions. These findings complement previous research on the metacognitive model of suicidality (12–14) as well as earlier evidence on the association between metacognitions and suicidal ideation (21–23). Notably, the relationships between suicide-related metacognitions and suicide-specific rumination were demonstrated for the first time in a non-Western sample of Iranian inpatients. This implies that the different variables are significant in understanding the escalation of suicidality across cultures. Against the backdrop of pronounced cultural differences of a general nature (46) and with regard to culture-related differences in general attitudes toward suicide (36), this finding cannot be taken for granted. Additionally, these associations remained significant even after controlling for lifetime suicide attempts, suicidal ideation, and depression (cf. 12–14). This suggests that they may represent a potentially important and cross-culturally relevant factor in the escalation of suicidality, warranting further investigation. It should be noted, however, that the present study did not test the assumption that suicide-specific rumination and negative suicide-related metacognitions contribute to the escalation of suicidal crises, i.e., that suicidal behavior becomes more likely as a result of the processes outlined here. In fact, separate logistic regression analyses revealed an association between suicide-specific rumination, negative suicide-related metacognitions, and suicide attempts, consistent with findings from two German studies on the metacognitive model of suicidality (12, 13), as well as additional studies in which suicide attempters and non-attempters differed with respect to general negative metacognitions concerning the uncontrollability and dangers of repetitive negative thinking (24, 25). However, a multiple logistic regression analysis showed no association between suicide-specific rumination, negative metacognitions, and lifetime suicide attempts in the current study. Rather, there was a positive association between suicidal ideation and a lifetime suicide attempt and a negative association between positive metacognitions and suicide attempts. This might indicate that positive metacognitions are more relevant for our understanding of suicidal ideation, whereas they might be less relevant to suicidal behavior (cf. 47). We would nonetheless caution against drawing far-reaching conclusions at this point. What is needed are future longitudinal studies to explore this aspect of the model in greater depth. In a similar vein, the present indications that diagnostic groups may differ with respect to suicide-specific rumination and suicide-related metacognitions should be replicated in larger samples before more far-reaching conclusions are drawn.

Given the consistent findings from the initial studies on the metacognitive model (12–14), the urgent need for new and innovative methods to treat suicidal patients (48), and the established effectiveness of metacognitive therapy (18), it seems timely to conduct a preliminary case study or case series using metacognitive therapy to treat suicidal patients (cf. 49, 50). This treatment could be based on the metacognitive manual for the treatment of depressed patients (15): Specifically, the treatment should include the following steps: case conceptualization, Attention Training Technique (ATT), detached mindfulness, cognitive modification of positive and negative suicide-related metacognitions as well as relapse prevention. To increase patient safety, these standard strategies of metacognitive therapy should be complemented by a prior safety plan intervention and means restriction counseling (51, 52). To date, there has been a particular lack of brief, suicide-specific interventions that have proven effective in the context of inpatient treatment (53). From the patient’s perspective, such empirically validated treatment options are long overdue; furthermore, they would also offer clinicians additional protection against legal malpractice disputes (54). However, whether and to what extent metacognitive therapy for suicidal patients is suitable for this purpose remains an open question at present.

Several limitations have to be considered when interpreting the results. First, due to the cross-sectional design of the current study, conclusions on causality have to be handled carefully. Second, only single aspects of the metacognitive model were examined in the current study; as such, attentional fixation and thought suppression were not taken into account (cf. 15). It must therefore be emphasized that the present study does not test the validity of the entire model, but only examined individual paths of the model. Caution should therefore be exercised when drawing far-reaching conclusions regarding the validity of the metacognitive model of suicidality. Third, the study relies exclusively on self-report measures, which is associated with methodological problems such as common-method variance, potential overlap and/or redundancy between items measuring suicidal ideation, suicide-specific rumination, and suicide-related metacognitions, misclassification of self-injurious behavior (cf. 55, 56), as well as difficulties in providing information about potentially automatic metacognitive processes (cf. 57). Fourth, the limited sample size precluded a comprehensive test of the entire model using, e.g., structural equation modelling, which could be subject to future studies using larger samples. Finally, it should be emphasized once again that, particularly in light of the limited sample size, comparisons between the different diagnostic groups must be regarded as purely exploratory and hypothesis-generating.

To conclude, the present study provides further evidence suggesting that the metacognitive model of suicidality might be a valid and helpful model to conceptualize and understand suicidal escalations. Therefore, prospective and high-resolution studies regarding the model’s assumptions as well as case studies on metacognitive therapy with suicidal patients seem to be warranted.

## Data Availability

The raw data supporting the conclusions of this article will be made available by the authors, without undue reservation.
